# DNA Barcodes and Morphology Reveal Five New Species of *Phanerotoma* (Hymenoptera, Braconidae, Cheloninae) from China

**DOI:** 10.3390/insects17020219

**Published:** 2026-02-20

**Authors:** Yu Fang, Wenjuan Luo, Cornelis van Achterberg, Xuexin Chen, Pu Tang

**Affiliations:** 1 State Key Lab of Rice Biology and Breeding, Zhejiang University, Hangzhou 310058, China; fang_yu1218@163.com (Y.F.); 17277631868@163.com (W.L.); xxchen@zju.edu.cn (X.C.); 2 Zhejiang Provincial Key Laboratory of Biology and Ecological Regulation of Crop Pathogens and Insects, Zhejiang University, Hangzhou 310058, China; 3 Ministry of Agriculture and Rural Affairs, Key Laboratory of Molecular Biology of Crop Pathogens and Insects, Zhejiang University, Hangzhou 310058, China; 4 Institute of Insect Sciences, College of Agriculture and Biotechnology, Zhejiang University, Hangzhou 310058, China; kees@vanachterberg.org

**Keywords:** DNA barcode, key, new species, phylogeny, taxonomy

## Abstract

The genus *Phanerotoma* Wesmael, 1838 (Hymenoptera, Braconidae, Cheloninae, Phane- rotomini) is distributed across all six major zoogeographical regions, with the highest species diversity recorded in the Palaearctic Region. DNA barcoding provides a robust method for species identification, yet its effectiveness for the genus *Phanerotoma* is limited by the scarcity of reliable, species-level data from specific regions in public databases. This gap makes it essential to contribute comprehensive genetic resources to advance taxonomic research. This study presents a comprehensive COI dataset of 92 sequences for the genus *Phanerotoma*, employing both the Automatic Barcode Gap Discovery (ABGD) method for species delimitation and the bPTP model for phylogenetic inference. The integrated analytical approach revealed 18 distinct species, including five new species; all species new to science are described and illustrated, and updates of the most recent key to the Chinese species are included.

## Simple Summary

The genus *Phanerotoma* Wesmael, 1838 comprises important parasitoid natural enemies of lepidopteran pests. Currently, approximately 300 species of *Phanerotoma* have been reported worldwide. However, the lack of host information often leads to the inability of traditional taxonomic methods to distinguish some cryptic species, thus making it essential to incorporate DNA barcoding for species identification. In the present study, analysis of 92 COI sequences combined with morphological evidence led to the discovery of five new *Phanerotoma* species in China.

## 1. Introduction

The taxonomic history of the genus *Phanerotoma* Wesmael, 1838 can be traced back to the early 19th century. The type species of this genus was first discovered by Panzer in 1805 and initially placed in the genus *Chelonus* [1]. However, it was not until 1838 that Wesmael formally established the genus *Phanerotoma* [2]. Throughout the late 19th and mid-20th centuries, the taxonomy of the genus advanced slowly, with the unstable generic concept subject and numerous redundant species descriptions. By the late 20th century, significant advancements were made by Zettel and van Achterberg. Zettel from 1988 to 1992 conducted comprehensive surveys across all six zoogeographic regions, describing 60 new species and substantially expanding the known distribution patterns of the genus [3,4,5,6,7,8,9,10,11,12,13,14]. Van Achterberg performed a thorough revision of the European fauna in 1990, establishing a standardized descriptive system that integrated type specimen information, ratios of fore wing venation, and multi-view morphological characters of the metasomal carapace [15]. In 2021, van Achterberg further identified key diagnostic characters, including the ratio of the mandibular teeth and morphological differences in the female hypopygium [16]. His terminological system has since become the international standard for taxonomic studies of this genus. Recently, Luo et al. confirmed the taxonomic status of 24 new species within the genus, discovered 6 newly recorded species for China, proposed 1 new synonym, introduced 1 new name, reinstated 1 species, and conducted a systematic revision of the Chinese species [17].

The genus *Phanerotoma* is distributed across all six major zoogeographical regions, with 168 species recorded in the Palaearctic, 23 in the Nearctic, 38 in the Neotropical, 32 in the Oriental, 38 in the Australian, and 41 in the Afrotropical regions [18]. Owing to the connectivity between several regions, particularly the Palaearctic and Oriental and the Palaearctic and Afrotropical, many species occur across two adjacent zoogeographical regions. In contemporary taxonomy, the integration of DNA barcoding as a diagnostic character has become increasingly common in the description of new species. China, which spans both the Palaearctic and Oriental regions, previously documented 46 known species of *Pha-nerotoma*. However, prior to this study, as in the revision by Luo et al. [17], the majority of these species were characterized solely by morphological descriptions, with a notable lack of reliable, species-level molecular data for this genus. To address this gap, our study provides over 90 DNA barcodes for *Phanerotoma*. Utilizing this molecular dataset, we identified and described five new species that were previously unrecognized cryptic species in China. These discoveries, enabled by the integrative approach, represent a distinct contribution that complements the morphological baseline. Specifically, our work provides the first substantial species-level COI barcode resource for Chinese *Phanerotoma*, offering independent data to test and refine the existing morphological hypotheses. This contribution not only enhances the taxonomic understanding of the genus in the region but also establishes a foundational molecular resource for future phylogenetic and species delimitation studies.

## 2. Materials and Methods

### 2.1. Taxon Sampling

The specimens examined in this study were collected through using sweep nets, light, and Malaise traps. All the specimens examined in this study are deposited in the Parasitic Hymenoptera Collection of Zhejiang University, Hangzhou, China (ZJUH).

### 2.2. Specimen Examination

Morphological descriptions adhered to the terminology and measurement standards established by van Achterberg [19,20] and He et al. [21]. Specimens were examined and measured using a Nikon SMZ800N stereoscopic microscope and illustrated with a KEYENCE VHX-7000 digital microscope (Osaka, Japan). The photos were composed and enhanced with Adobe Photoshop 2023. The following abbreviations are used: OOL = shortest distance from a posterior ocellus to nearest eye margin; OD = maximum diameter of posterior ocellus; POL = minimum width between posterior ocelli.

### 2.3. DNA Extraction, PCR Amplification and Sequencing

Genomic DNA was extracted non-destructively using the QIAamp DNA Mini Kit (Qiagen, Hilden, Germany) [22], with sequence data provided in Table S1. Amplification of the approximate 658 bp COI barcode region [23] employed primers LCO1490 and HCO2198 [24] and the KOD One™ PCR Master Mix. The PCR program consisted of an initial 5 min at 98 °C, followed by five cycles of 30 s at 98 °C, 40 s at 45 °C, and 1 min at 72 °C; then, 35 cycles of 30 s at 98 °C, 40 s at 55 °C, and 1 min at 72 °C, with a final 5 min extension at 72 °C.

### 2.4. Data Analyses

The PCR products were sequenced in both forward and reverse directions, and the resulting reads were assembled and edited in Geneious Prime 2024.0.5. For comparative analysis, 42 congeneric sequences were obtained from BOLD Systems v3, and one outgroup sequence was retrieved from NCBI (both on 12 December 2025; see Table S1). All sequences were translated to amino acids within Geneious to screen for stop codons, followed by alignment using MAFFT v7.505 [25]. The final alignment was 729 bp in length, containing undefined nucleotides (N) for some sequences.

Genetic distances (intra- and interspecific K2P pairwise distances) were computed in MEGA-X [26] (Table S2). Maximum-likelihood (ML) phylogenies were inferred using IQ-TREE v2.1.3 [27] with the best-fit substitution model selected by Model Finder (MFP). The phylogenetic trees were visualized and illustrated using tvBOT (https://www.chiplot.online/tvbot.html (accessed on 19 December 2025)) [28].

Species delimitation employed two complementary methods: the distance-based Automatic Barcode Gap Discovery (ABGD) and the tree-based Poisson Tree Process (PTP) [29]. The former partitions sequences into candidate species by automatically detecting the barcode gap between intra- and interspecific variation, without requiring a priori thresholds [30]. ABGD analysis was conducted via the iTaxoTools web interface (https://itaxotools.org/abgd.zip, accessed on 14 December 2025) using the K2P model, with a relative gap width (X) of 1.0, 15 threshold steps, and other parameters at default. The bPTP analysis was performed on the web (https://species.h-its.org/ptp/, accessed on 14 December 2025) with an unrooted tree type, 100,000 MCMC generations, and default settings for remaining parameters.

## 3. Results

### 3.1. COI Sequence Analysis

**Figure 1:**
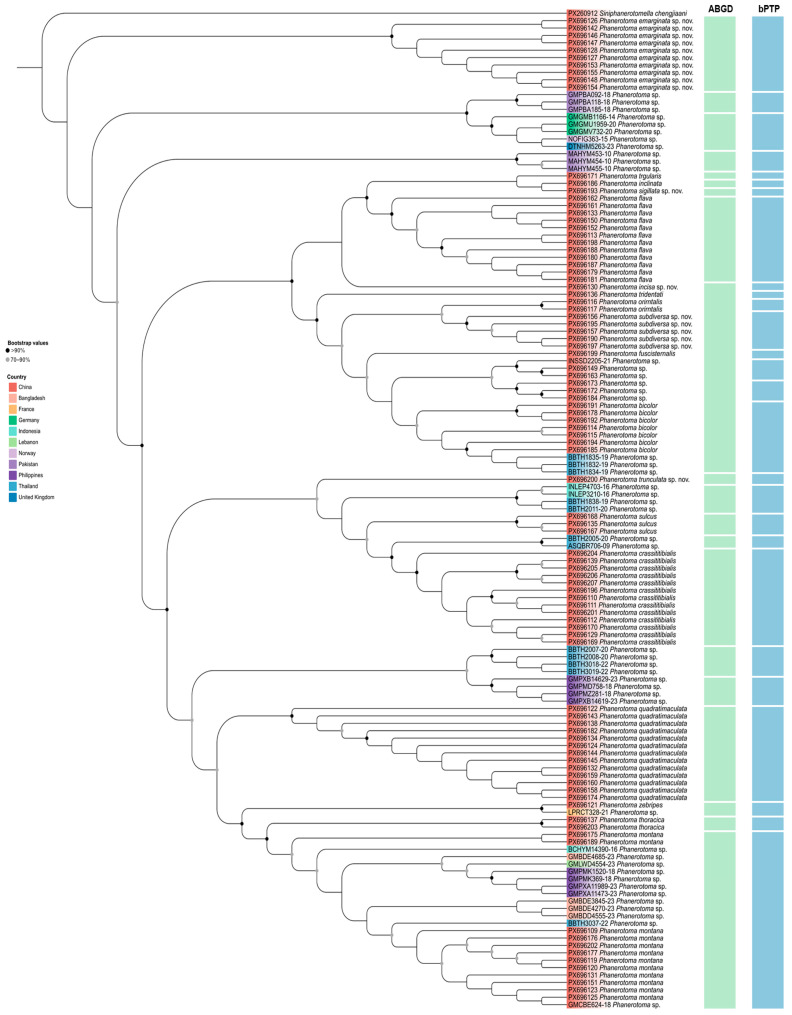
ML phylogenetic tree, based on 136 COI sequences highlighting the results of two delimitation analyses in *Phanerotoma.* The results of delimitation analyses are displayed with the vertical bars corresponding to putative species (MOTUs) inferred by ABGD and bPTP methods.

A DNA barcoding dataset was assembled for analysis, comprising 92 newly gene-rated sequences and an additional 42 sequences retrieved from public databases. Molecular operational taxonomic units (MOTUs) were delimited using two independent methods. The ABGD analysis delineated the sequences into 19 primary MOTUs. In contrast, the bPTP analysis, which incorporates phylogenetic relationships, suggested a finer partition, resulting in 27 MOTUs (Figure 1). In subsequent integrative taxonomic assessment, comparative morphology of voucher specimens provided stronger support for the more differentiated grouping recovered by bPTP. Based on congruent genetic and diagnostic morphological evidence, five of the delimited MOTUs are proposed as putative new species: *Phanerotoma emarginata* sp. nov., *P. incisa* sp. nov., *P. sigillata* sp. nov., *P. subdiversa* sp. nov., and *P. trunculata* sp. nov.

The Kimura-2-parameter (K2P) genetic distances of interspecies and intraspecies are summarized in Table S2; genetic distance analyses revealed barcoding gaps between intra- and interspecific variation. The interspecific distances ranged from 2.8% (with the lowest observed between BBTH1834-19 *Phanerotoma bicolor* and PX696172 *Phanerotoma* sp.) to 19.5% (with the highest observed between GMPXA11989-23 *Phanerotoma montana* and PX696127 *Phanerotoma emarginata* sp. nov.).

### 3.2. Taxomomy

Genus *Phanerotoma* Wesmael, 1838

*Phanerotoma* Wesmael, 1838: 165 [2]. Type-species: *Chelonus dentatus* Panzer, 1805. Designated by Haliday, 1840: 63 [31]; Shenefelt, 1973: 909 [32].

Diagnosis. Antenna with 23 segments in both sexes (except 25–27 segments in *P. potanini*). FEMALE. Eyes glabrous. Clypeus with 2–3 ventral medium-sized to minute teeth or teethless. Fore wing pterostigma often relatively robust; vein 1-SR+M present; vein 2-R1 absent; second submarginal cell broad or slender; first discal cell more or less truncate anteriorly; vein CU1b developed, resulting in a closed first subdiscal cell. Hind wing usually with vein r present; vein M+CU equal to or longer than vein 1-M. Metasomal carapace with two distinct transverse sutures; third tergite without lateral teeth or at most with angulate projection posterolaterally. At most one distal third of ovipositor sheath setose.

Host. Lepidoptera larvae.

Distribution. Worldwide.

### 3.3. Descriptions of New Species

#### 3.3.1. *Phanerotoma emarginata* Fang, Luo and van Achterberg, sp. nov. (Figure 2)

Zoobank: urn:lsid:zoobank.org:act:C2525624-B835-4441-A56E-B34410201686

**Figure 2:**
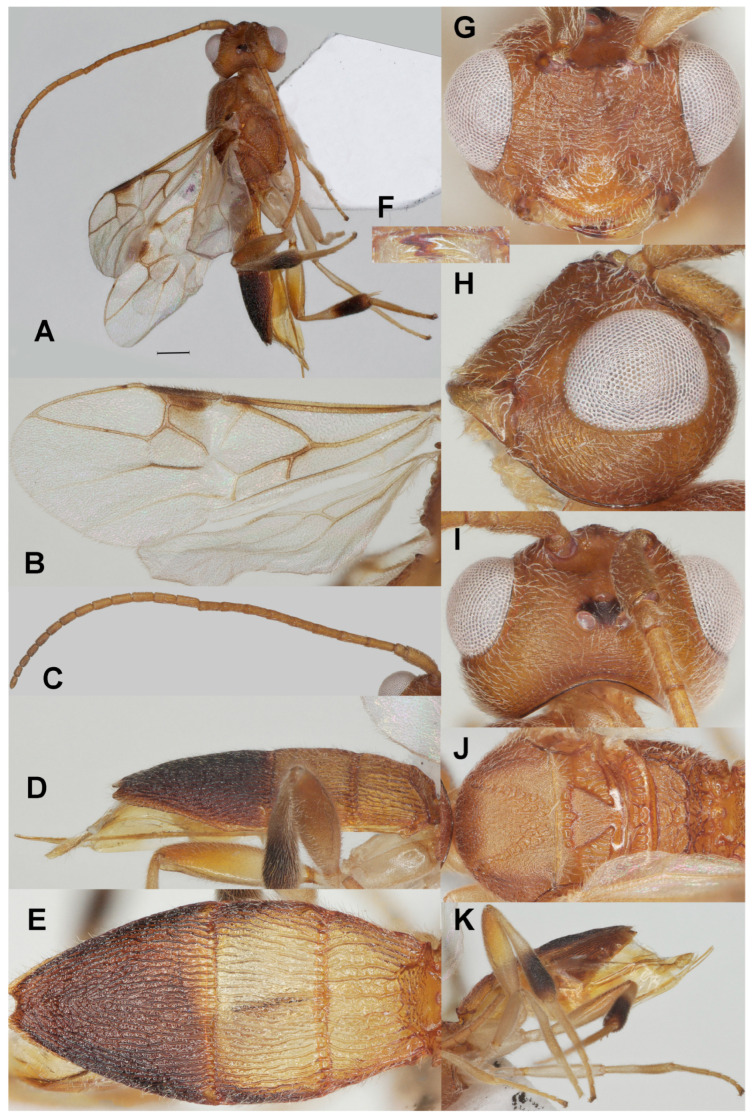
*Phanerotoma emarginata* sp. nov., holotype, ♀. (ZJUH No. 202402503) (**A**) Habitus, lateral aspect. (**B**) Wings. (**C**) Antenna, lateral aspect. (**D**) Metasoma, lateral aspect. (**E**) Metasoma, dorsal aspect. (**F**) Mandible, ventral aspect. (**G**) Head, anterior aspect. (**H**) Head, lateral aspect. (**I**) Head, dorsal aspect. (**J**) Mesosoma, dorsal aspect. (**K**) Hind leg, lateral aspect. Scale bar: 1.0 mm.

Diagnosis. Clypeus without distinct ventral teeth (Figure 2G); subapical antennal segments of ♀ non-moniliform, cylindrical (Figure 2C); temple weakly convex dorsally in lateral view (Figure 2H); malar space 0.8× as long as basal width of mandible (Figure 2H); vein 2-SR of fore wing slightly curved and diverging from posterior margin of pterostigma (Figure 2B); length of 1-R1 of fore wing 1.6× as long as pterostigma (Figure 2F); lateral sides of third tergite of metasoma in dorsal view straight and directly narrowing posteriorly, nearly triangular (Figure 2E); prolongation of hypopygium of ♀ slender and much longer than its basal width (Figure 2K); in lateral view metasomal carapace rather flat and thin, medio-posteriorly with semicircular emargination (Figure 2D).

Runs in the key to Chinese *Phanerotoma* species by Luo et al. [17] to couplet 4 and can be inserted as follows:

4 Lateral sides of third tergite of metasoma in dorsal view straight and directly narrowing posteriorly, nearly triangular (Figure 26G in Luo et al. [17]); in lateral view, metasomal carapace rather flat and thin, but with triangular convex area posteriorly (Figure 26H l.c.); 4–5 subapical segments of ♀ antenna moniliform (Figure 26J l.c.), except in *P. emarginata* ··································································································5a

- Lateral sides of third tergite of metasoma in dorsal view rounded and gradually narrowing posteriorly (Figure 28G l.c.); in lateral view, metasomal carapace more convex and thick (Figure 28H l.c.); if rather flat, then no triangular convex area posteriorly (Figure 31C l.c.); subapical segments of ♀ antenna often not moniliform (Figure 31F l.c.)····························································································6

5a Third tergite of ♀ with small semicircular emargination medio-posteriorly (Figure 2E,K), with very weak depression in ♂; metasoma strongly flattened (Figure 2D); subapical segments of ♀ antenna not moniliform (Figure 2C); [prolongation of hypopygium of ♀ slender and much longer than its basal width (Figure 2K)]········································································································*P. emarginata* sp. nov.

- Third tergite of ♀ rounded or truncate medio-posteriorly (Figures 5H and 26G in Luo et al. [17]); metasoma at least weakly convex; subapical segments of ♀ antenna moniliform (Figure 26J l.c.)········································································································5

Materials examined. Holotype, ♀, China: Shaanxi, Danfeng, 10 August 2010, light trap, (ZJUH No. 202402503). Paratypes, 8♀♀, 1♂, China: Shaanxi, Danfeng, 10 August 2010, light trap, (ZJUH Nos. 202402504, 202402507, 202402540, 202402542, 202402543, 202402549–202402551, 202402519).

Description. Holotype. ♀ (Figure 2). Length of body 5.0 mm, fore wing 3.5 mm.

Color. Yellowish brown; stemmaticum, mandible apically, veins r, 2-SR, 3-SR, 2-M, 1-CU1, and cu-a of fore wing, pterostigma (except pale base) and parastigma of fore wing, second tergite laterally, third tergite and apical half of hind tibia dark brown; hind tibia basally ivory; antenna apically and patch below pterostigma darkened; vein 1-M of fore wing brownish yellow or brown; remainder of legs pale yellowish, but hind femur brownish apico-dorsally; tegula yellow and humeral plate dark brown.

Head (Figure 2C,F–I) Width 1.4× median length in anterior view (Figure 2G) and part of head above eye in lateral view 0.3× height of eye (Figure 2H); antenna with 23 segments and 1.3× as long as fore wing, subapical antennal segments non-moniliform, cylindrical (Figure 2C), third, fourth and penultimate segments 3.0, 3.0 and 1.5× as long as wide in lateral view; area of stemmaticum mainly granulate; OOL: diameter of posterior ocellus: POL = 3:1:1; eye 1.5× temple in dorsal view (Figure 2I); frons with curved rugae medially, rugose laterally and without median carina; vertex transversely rugulose-striate and with satin sheen; temple largely superficially striate and rather shiny; face transversely or obliquely rugose and without distinct median ridge; clypeus mostly smooth, shiny and 0.7 times as wide as minimum width of face, intertentorial distance 1.2× minimum width between clypeus and eye, clypeus with medium-sized setae and no distinct teeth medio-ventrally (Figure 2G), at most with two hardly visible protruding corners; eye in lateral view 1.2× as wide as temple (Figure 2H); in anterior view height of eye 0.7× minimum width of face; upper condyle of mandible below lower level of eyes (Figure 2G); malar space 0.8× as long as basal width of mandible; lower tooth of mandible 0.4× length of apical tooth, robust (Figure 2F).

Mesosoma (Figure 2J). Length 1.4× its width in lateral view; side of pronotum mainly crenulate medially, and remainder nearly smooth; mesoscutum mainly finely granulate (but medio-posteriorly rugose) and with satin sheen, densely setose; notauli clearly indicated, crenulate; scutellar sulcus rather wide and with six carinae (Figure 2J); scutellum triangular, flat, finely granulate and rather shiny; metanotum with short median carina anteriorly and no tooth protruding posteriorly; propodeum coarsely reticulate-rugose, with transverse carina, no median carina, latero-posteriorly tuberculate.

Wings (Figure 2B). Fore wing 2.9× as long as its maximum width; length of 1-R1 1.5× as long as pterostigma; r issued rather far beyond middle of pterostigma and 0.9× as long as 3-SR; distance between 1-R1 and wing apex 0.3× 1-R1; 2-SR straight but slightly curved posteriorly and diverging from posterior margin of pterostigma (Figure 2B); SR1 straight; parastigma medium-sized; m-cu interstitial; 1-CU1 0.4× as long as vein 2-CU1, cu-a moderately inclivous and 0.9× as long as 3-CU1; r:3-SR:SR1 = 10:11:65; 2-SR:3-SR:r-m = 30:11:13; 2-M weakly curved (Figure 2B).

Legs (Figure 2K). Hind femur rather dull, very finely sculptured, 4.0× as long as wide and rather widened submedially; hind tibia rather swollen (Figure 2K); middle tibia with small pale yellowish blister; hind coxa superficially granulate and shiny.

Metasoma (Figure 2D,E,K). Carapace elliptical in dorsal view, 2.2× as long as wide and 1.3× as long as mesosoma; first and second tergites coarsely and densely longitudinally rugose; second suture rather narrow; third tergite 2.0× as long as second tergite and laterally straight, strongly flattened in lateral view, in dorsal view triangular, densely and coarsely rugose and apically with shallow V-shaped emargination (Figure 2E); ovipositor sheath narrow and parallel-sided, with some long and erect setae; prolongation of hypopygium slender and much longer than its basal width (Figure 2K).

Variation. Length of fore wing of female 3.5–4.1 mm, of male 3.0 mm; third tergite 1.9–2.2× as long as second tergite, curved to nearly straight laterally; POL 0.8–1.0× as long as diameter of posterior ocellus; eye medium-sized and in lateral view 1.2–1.4× as wide as temple; vein 2-SR of fore wing straight to slightly sinuate.

Male. Very similar to female, but third tergite without distinct medio-apical emargination and less pointed in dorsal view; hind femur yellowish brown.

Host. Unknown.

Distribution. China (Shaanxi).

Etymology. Named after the small semicircular emargination of the third metasomal tergite, from the Latin ‘*emarginatus*’ for ‘notched at the apex’.

#### 3.3.2. *Phanerotoma incisa* Fang, Luo and van Achterberg, sp. nov. (Figure 3)

Zoobank: urn:lsid:zoobank.org:act:C36AD506-57DE-4721-9942-331AA9062490

**Figure 3:**
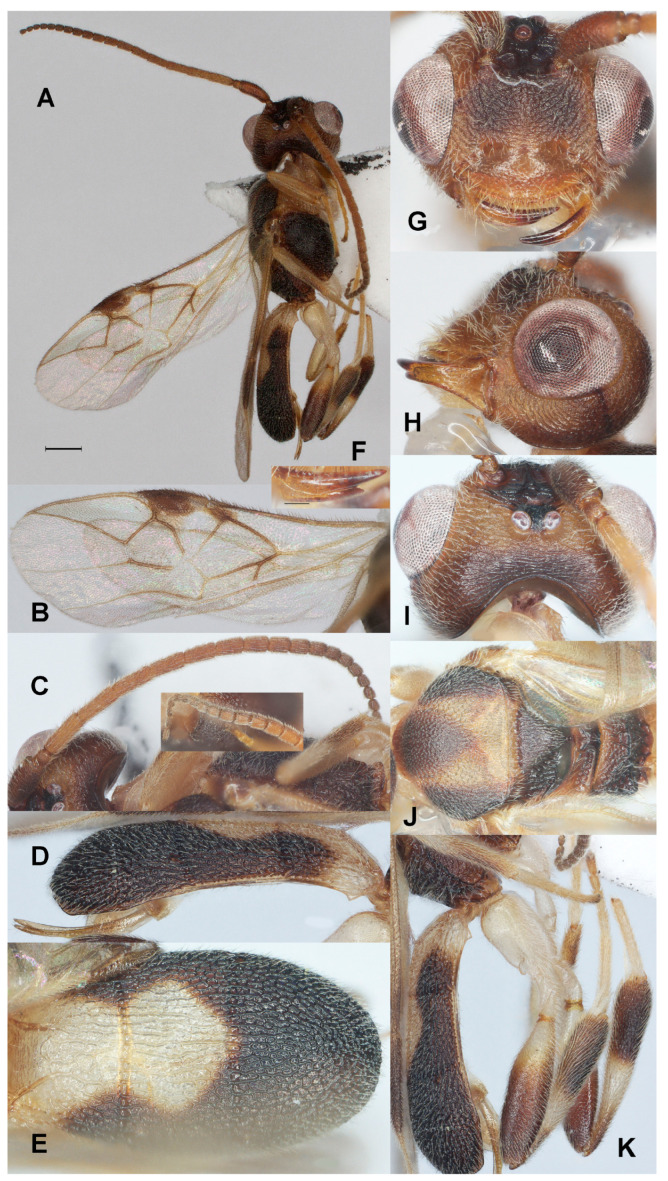
*Phanerotoma incisa* sp. nov., holotype, ♀. (ZJUH No. 202402509) (**A**) Habitus, lateral aspect. (**B**) Wings. (**C**) Antenna, lateral aspect. (**D**) Metasoma, lateral aspect. (**E**) Metasoma, dorsal aspect. (**F**) Mandible, ventral aspect. (**G**) Head, anterior aspect. (**H**) Head, lateral aspect. (**I**) Head, dorsal aspect. (**J**) Mesosoma, dorsal aspect. (**K**) Hind leg, lateral aspect. Scale bar: 1.0 mm.

Diagnosis. Clypeus with three acute small ventral teeth (Figure 3G); nine subapical antennal segments of ♀ moniliform and narrowed basally (Figure 3C); temple convex dorsally in lateral view (Figure 3H); malar space 0.4× as long as basal width of mandible (Figure 3H); vein 2-SR of fore wing rather bent and diverging from posterior margin of pterostigma (Figure 3B); length of 1-R1 of fore wing 1.3× as long as pterostigma (Figure 3B); lateral sides of third tergite of metasoma in dorsal view curved and rounded posteriorly (Figure 3E); apical prolongation of hypopygium of ♀ short and lateral sides about equal to its basal width; in lateral view metasomal carapace moderately convex, medio-posteriorly shallowly concave in posterior view.

Runs in the key to Chinese *Phanerotoma* species by Luo et al. [17] to couplet 4 and can be inserted as follows:

42 Hypopygium acute apically, either with medium-sized and slender triangular protuberance (Figure 13J in Luo et al. [17]) or with short triangular protuberance; apical half of hind femur dark brown (Figure 13F l.c.); clypeus with three small but distinct teeth medio-ventrally (Figure 13E l.c.); pterostigma dark brown (Figure 13G l.c.); lower tooth of mandible 0.2–0.3 times as long as apical tooth (Figure 13L l.c.) ································································································43a

- Hypopygium truncate, without triangular apex (Figures 16J and 25M l.c.); apical half of hind femur yellow (Figures 16K and 25L l.c.); clypeus with three minute teeth (Figures 16E and 25E l.c.); pterostigma brown (Figures 16G and 25H l.c.); lower tooth of mandible 0.5 times as long as apical tooth (Figures 16L and 25N l.c.); [third tergite of metasoma rounded posteriorly (Figures 16I and 25J l.c.)] ································································································43

43a Antenna of ♀ with about 5 subapical moniliform segments (Figure 13F l.c.); third tergite of metasoma truncate posteriorly (Figure 13I l.c.); acute apex of hypopygium comparatively long (Figure 13K l.c.); emargination of head shallower (Figure 13C l.c.); hind tibia of ♀ wide (Figure 13K l/c.) ································································································*P. fuscisternalis* Luo et al., 2025

- Antenna of ♀ with about 9 subapical moniliform segments (Figure 3C); third tergite of metasoma rather rounded posteriorly in dorsal view (Figure 3E); acute apex of hypopygium short and equilateral; emargination of head deep (Figure 3H); hind tibia of ♀ less wide (Figure 3J) ································································································*P. incisa* sp. nov.

Materials examined. Holotype, ♀, China: Shaanxi, Danfeng, 10 August 2010, light trap, (ZJUH No. 202402509).

Description. Holotype. ♀ (Figure 2). Length of body 4.8 mm, fore wing 3.8 mm.

Color. Dark brown; vertex (except stemmaticum and vertex posteriorly), frons medially, temple (except posteriorly), face laterally and ventro-medially, clypeus, malar space, mandible (except apex), veins of hind wing and basal third of fore wing, vein 1-R1 of fore wing, tegula largely, large medial patch of mesoscutum, pronotum, fore and middle legs (except apical half of tibiae dorsally), hind leg (but femur only basally, tibia only subbasal band, first metasomal tergite (except posterior corners), and large patch of second tergite pale yellowish to yellowish brown; pterostigma (except small pale base) and parastigma of fore wing dark brown; antenna apically, scape and patch below pterostigma darkened; vein 1-M of fore wing mainly dark brown.

Head (Figure 3C,F–I) Width 1.3× median length in anterior view (Figure 3G) and part of head above eye in lateral view 0.3× height of eye (Figure 3H); antenna with 23 segments and 1.1× as long as fore wing, nine subapical antennal segments moniliform, narrowed basally (Figure 3C), third, fourth and penultimate segments 3.0, 2.7 and 1.7× as long as wide in lateral view; area of stemmaticum mainly granulate; OOL: diameter of posterior ocellus: POL = 26:10:5; eye protruding, 1.5× temple in dorsal view (Figure 3I); frons with some curved rugae medially, rugose lateral with oblique rugae and without median carina; vertex finely granulate and posteriorly finely transversely rugulose-striate; temple largely with curved striae; face coarsely rugose and without distinct median ridge; clypeus mostly smooth, superficially punctulate, shiny and about as wide as minimum width of face, intertentorial distance 2.5× minimum width between clypeus and eye, clypeus with medium-sized setae and three acute teeth medio-ventrally (Figure 3G); eye in lateral view 1.6× as wide as temple (Figure 3H), in anterior view height of eye 0.85× minimum width of face; upper condyle of mandible below lower level of eyes (Figure 3G); malar space 0.4× as long as basal width of mandible; lower tooth of mandible 0.4× length of apical tooth, robust (Figure 3F); head deeply emarginate medio-posteriorly (Figure 3I).

Mesosoma (Figure 3J). Length 1.5× its width in lateral view; side of pronotum mainly crenulate medially, and remainder rugose, shiny; mesoscutum mainly densely rugulose (but medio-posteriorly rugose) and with satin sheen, densely setose; notauli weakly indicated, rugose; scutellar sulcus rather narrow and with four carinae (Figure 3J); scutellum triangular, weakly convex, finely rugulose; metanotum with short median carina anteriorly and no tooth protruding posteriorly; propodeum coarsely reticulate-rugose, with transverse carina, no median carina, latero-posteriorly tuberculate.

Wings (Figure 3B). Fore wing 2.7× as long as its maximum width; length of 1-R1 1.3× as long as pterostigma; r issued rather far beyond middle of pterostigma and 0.2× as long as 3-SR; distance between 1-R1 and wing apex 0.2× 1-R1; 2-SR distinctly bent and diverging from posterior margin of pterostigma (Figure 3B); SR1 straight or nearly so; parastigma medium-sized; m-cu slightly antefurcal; 1-CU1 0.3× as long as vein 2-CU1, cu-a moderately inclivous and about as long as 3-CU1; r:3-SR:SR1 = 2:10:27; 2-SR:3-SR:r-m = 3:2:1; 2-M weakly curved (Figure 3B).

Legs (Figure 3K). Hind femur rather dull, very finely sculptured, 3.4× as long as wide and rather widened submedially; hind tibia swollen (Figure 3K); middle tibia with rather small whitish blister; hind coxa superficially granulate and shiny.

Metasoma (Figure 3D,E,K). Carapace broad elliptical in dorsal view, 1.8× as long as wide and about as long as mesosoma; first and second tergites coarsely and densely longitudinally rugose; second suture rather narrow; third tergite 1.3× as long as second tergite and laterally curved, distinctly convex in lateral view, in dorsal view rounded, densely and coarsely rugose and medio-posteriorly shallowly concave in posterior view; ovipositor sheath comparatively wide (Figure 3D) and parallel-sided, with some long and erect setae; prolongation of hypopygium short and lateral sides equal to its basal width.

Male. Unknown.

Host. Unknown.

Distribution. China (Shaanxi).

Etymology. Named after the deeply concave head in dorsal view (Figure 3I), from the Latin ‘*incisus*’ for ‘cut into’.

#### 3.3.3. *Phanerotoma sigillata* Fang, Luo and van Achterberg, sp. nov. (Figure 4)

Zoobank: urn:lsid:zoobank.org:act:5B5A084F-5543-453F-97CA-360436F33E81

**Figure 4:**
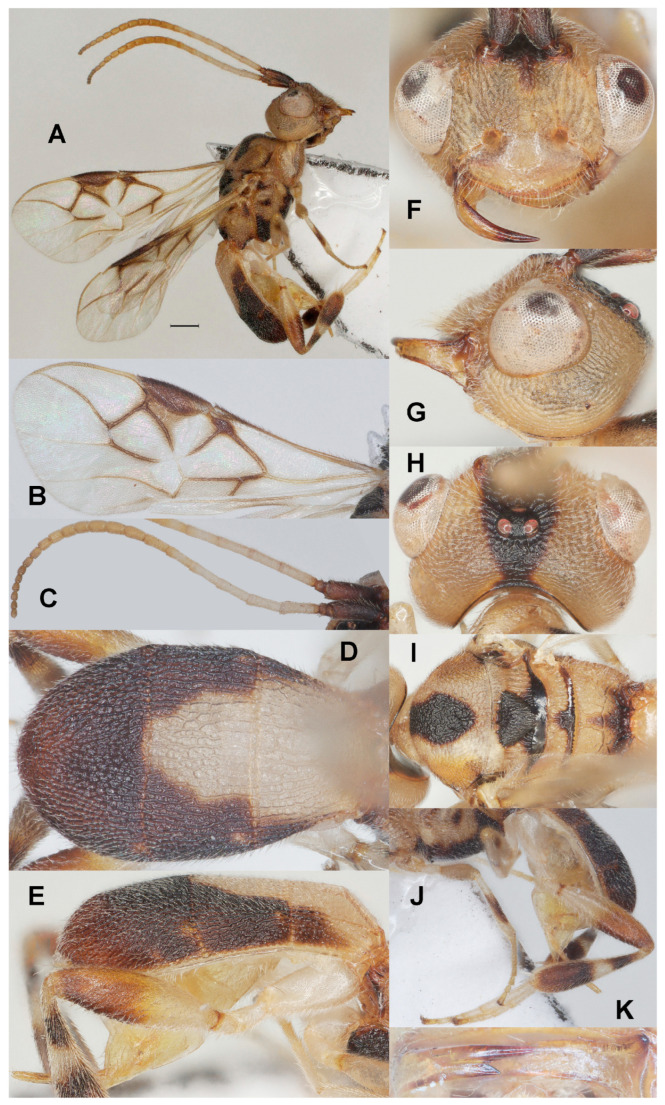
*Phanerotoma sigillata* sp. nov., holotype, ♀. (ZJUH No. 202502653) (**A**) Habitus, lateral aspect. (**B**) Fore wings. (**C**) Antenna, lateral aspect. (**D**) Metasoma, dorsal aspect. (**E**) Metasoma, lateral aspect. (**F**) Head, anterior aspect. (**G**) Head, lateral aspect. (**H**) Head, dorsal aspect. (**I**) Mesosoma, dorsal aspect. (**J**) Hind leg, lateral aspect. (**K**) Mandible, ventral aspect. Scale bar: 1.0 mm.

Diagnosis. Clypeus with three small ventral teeth between long setae (Figure 4F); eight subapical antennal segments of ♀ moniliform and narrowed basally (Figure 4C) and scape strongly contrasting with third antennal segment; temple truncate dorsally in lateral view (Figure 4G); eyes protuberant and 1.3 times longer than temple in dorsal view (Figure 4H); malar space 0.4× as long as basal width of mandible (Figure 4G); vein 2-SR of fore wing weakly curved and diverging from posterior margin of pterostigma and vein SR1 slightly curved (Figure 4B); length of 1-R1 of fore wing 1.1× as long as pterostigma (Figure 4B); lateral sides of third tergite of metasoma in dorsal view curved and rounded posteriorly (Figure 4D); apical prolongation of hypopygium of ♀ short and rather slender (Figure 4K); in lateral view metasomal carapace moderately convex, medio-posteriorly shallowly concave in posterior view; mesosoma and legs with pattern of dark brown marks (Figure 4A,I,J).

Runs in the key to Chinese *Phanerotoma* species by Luo et al. [17] to couplet 4 and can be inserted as follows:

18 Eye 1.1–1.3 times longer than temple in dorsal view (Figure 29C in Luo et al. [17]); third tergite of metasoma rounded posteriorly (Figure 29H l.c.); hind femur mostly dark brown (Figure 29J l.c.), at least in apical half darker than its basal half ································································································19a

- Eye 1.6–1.9 times longer than temple in dorsal view (Figure 38D l.c.); third tergite of metasoma truncate posteriorly (Figure 38I l.c.); hind femur mostly yellowish brown (Figure 38K l.c.) ································································································20

19a Antenna tricolored, scape and pedicel dark brown (contrasting with third segment), apical half yellow and remainder white (Figure 4C); body with complicated dark brown/pale yellowish pattern (Figure 4A,I); most veins of fore wing dark brown and their surroundings distinctly infuscated (Figure 4C); eight subapical segments of ♀ antenna moniliform (Figure 4C); [hind tarsus whitish] ································································································*P. sigillata* sp. nov.

- Antenna dark brown or brown; body and legs without complicated pattern; most veins of fore wing moderately darkened and their surroundings largely subhyaline; at most seven subapical segments of ♀ antenna moniliform ································································································19b

Materials examined. Holotype, ♀, China: Zhejiang, Mt. Xitianmu, Lieshuici, 23 Jun 2011, light trap, (ZJUH No. 202502653).

Description. Holotype. ♀ (Figure 4). Length of body 5.5 mm, fore wing 4.0 mm.

Color. Pale yellowish or ivory, basal half of antenna (except scape and pedicel) white and remainder pale brown; scape, pedicel, triangular medial mark of frons, vertex and occiput (Figure 4H), veins of medial third of fore wing, patch below pterostigma, humeral plate anteriorly largely, large medial patch of mesoscutum, scutellum up to metanotum, metanotum and propodeum posteriorly, ventrally mesosoma (except small medial patch), mesopleuron ventrally, pair of small patches dorsally, small patch on metapleuron, fore and middle femora subapical patch ventrally and tibiae medio-dorsally, subbasal band and apical half of hind tibia, posterior corners of first metasomal tergite, second tergite laterally and third tergite (except brownish apical part) dark brown; pterostigma (except small pale basal patch), vein 1-M and parastigma of fore wing dark brown; antenna apically, and apical half of hind femur darkened and more or less brown (Figure 4C,E); vein 1-R1 of fore wing yellow.

Head (Figure 4C,F–H,K) Width 1.3× median length in anterior view (Figure 4F) and part of head above eye in lateral view 0.3× height of eye (Figure 4G); antenna with 23 segments and 1.2× as long as fore wing, eight subapical antennal segments moniliform, narrowed basally (Figure 4C), third, fourth and penultimate segments 3.6, 3.0 and 1.3× as long as wide in lateral view; area of stemmaticum granulate; OOL: diameter of posterior ocellus: PÓL = 19:5:4; eye distinctly protruding, 1.3× temple in dorsal view (Figure 4H); frons rugose and without median carina; vertex rather coarsely rugose; temple largely with curved rugae; face coarsely rugose and with medio-dorsal protuberance (Figure 4F); clypeus smooth except superficial punctulation, shiny and about as wide as minimum width of face, intertentorial distance 1.6× minimum width between clypeus and eye, clypeus with long setae and three minute teeth medio-ventrally (Figure 4F); eye in lateral view about as wide as temple (Figure 4G), in anterior view height of eye 0.7× minimum width of face; upper condyle of mandible below lower level of eyes (Figure 4F); malar space 0.4× as long as basal width of mandible; lower tooth of mandible 0.3× length of apical tooth, robust (Figure 4K); head moderately emarginate medio-posteriorly (Figure 4H).

Mesosoma (Figure 4I). Length 1.5× its width in lateral view; side of pronotum coarsely crenulate medially, and remainder rugose, shiny; mesoscutum mainly densely and rather finely rugose (but medio-posteriorly coarsely rugose) and with satin sheen, densely setose; notauli shallowly impressed, crenulate (Figure 4I); scutellar sulcus rather wide and with five carinae (Figure 4I); scutellum triangular, weakly convex, granulate-rugulose; metanotum with medium-sized median carina anteriorly and no tooth protruding posteriorly; propodeum coarsely reticulate-rugose, with transverse carina, no median carina, latero-posteriorly not tuberculate.

Wings (Figure 4B). Fore wing 2.7× as long as its maximum width; length of 1-R1 1.1× as long as pterostigma; r issued rather far beyond middle of pterostigma and 0.2× as long as 3-SR; distance between 1-R1 and wing apex 0.2× 1-R1; 2-SR slightly curved and diverging from posterior margin of pterostigma (Figure 4B); SR1 curved; parastigma large; m-cu interstitial; 1-CU1 0.3× as long as vein 2-CU1, cu-a moderately inclivous and about as long as 3-CU1; r:3-SR:SR1 = 4:18:41; 2-SR:3-SR:r-m = 14:9:3; 2-M weakly curved (Figure 4B).

Legs (Figure 4J). Hind femur rather dull, very finely sculptured, 3.5× as long as wide and rather widened submedially; hind tibia swollen (Figure 4J); middle tibia with medium-sized whitish blister, but apically darkened; hind coxa superficially granulate and shiny.

Metasoma (Figure 4D,E,J). Carapace oval in dorsal view, 1.7× as long as wide and 1.1× as long as mesosoma, dorsal carinae of first tergite distinct; first and second tergites coarsely and densely longitudinally rugose; second suture rather narrow; third tergite 1.5× as long as second tergite and laterally curved, distinctly convex in lateral view, in dorsal view rounded, densely and coarsely rugose and medio-posteriorly shallowly concave in posterior view; ovipositor sheath comparatively wide (Figure 4J) and parallel-sided, with few short setae; prolongation of hypopygium short and rather slender (Figure 4K).

Male. Unknown.

Host. Unknown.

Distribution. China (Zhejiang).

Etymology. Named after the complicated mesosomal pattern of dark marks (Figure 4I), from the Latin ‘*sigillatus*’ for ‘adorned with marks’.

#### 3.3.4. *Phanerotoma subdiversa* Fang, Luo and van Achterberg, sp. nov. (Figure 5)

Zoobank: urn:lsid:zoobank.org:act:DBBF2ADB-7203-48E6-A2EF-FF7EB0EEA1F3

**Figure 5:**
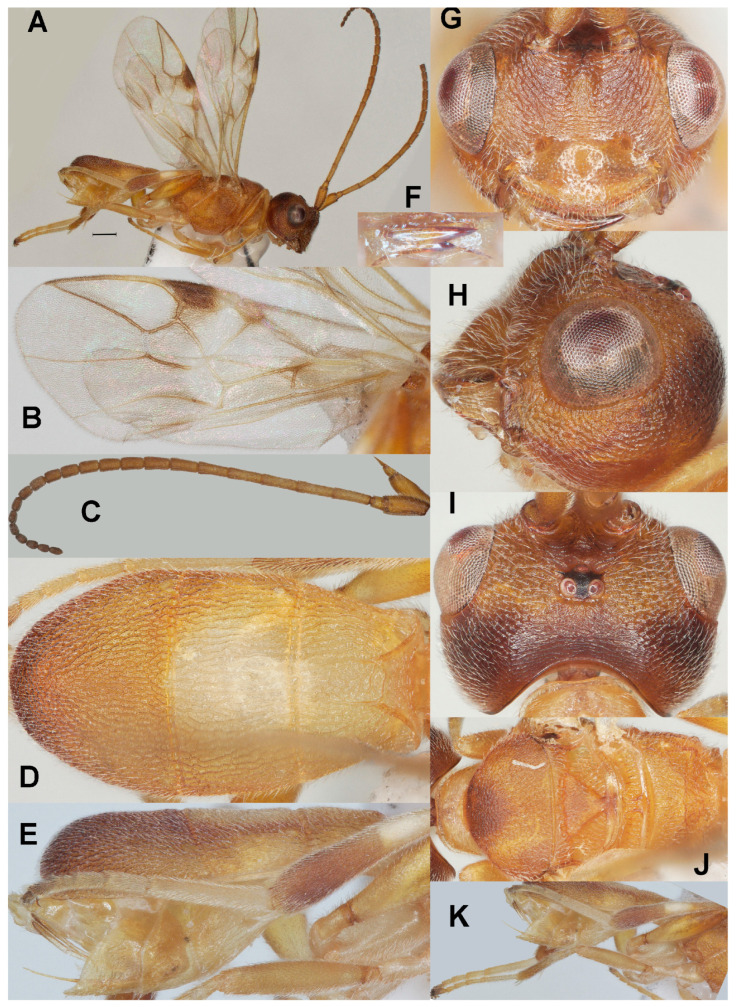
*Phanerotoma subdiversa* sp. nov., holotype, ♀. (ZJUH No. 202402509) (**A**) Habitus, lateral aspect. (**B**) Wings. (**C**) Antenna, lateral aspect. (**D**) Metasoma, dorsal aspect. (**E**) Metasoma, lateral aspect. (**F**) Mandible, ventral aspect. (**G**) Head, anterior aspect. (**H**) Head, lateral aspect. (**I**) Head, dorsal aspect. (**J**) Mesosoma, dorsal aspect. (**K**) Hind leg, lateral aspect. Scale bar: 1.0 mm.

Diagnosis. Clypeus with three acute medium-sized ventral teeth (Figure 5G); six subapical antennal segments of ♀ moniliform and narrowed basally (Figure 5C); temple convex dorsally in lateral view (Figure 5H); malar space 0.6× as long as basal width of mandible (Figure 5H); vein 2-SR of fore wing nearly straight and diverging from posterior margin of pterostigma (Figure 5B); length of 1-R1 of fore wing 1.3× as long as pterostigma, bicolored (Figure 5B); lateral sides of third tergite of metasoma in dorsal view curved and rounded posteriorly (Figure 5D); apical prolongation of hypopygium of ♀ short and lateral sides about equal to its basal width; in lateral view metasomal carapace moderately convex, medio-posteriorly shallowly concave in posterior view.

Runs in the key to Chinese *Phanerotoma* species by Luo et al. [17] to couplet 4 and can be inserted as follows:

19b Maximum width of pterostigma about 1.6 times length of vein 3-SR (Figure 29F in Luo et al. [17]); head dark brown or blackish and mesosoma yellow (Figure 29B,G l.c.); POL about 0.7 times diameter of posterior ocellus (Figure 29B l.c.); apical half of middle tibia brown (Figure 29E l.c.); apical half of antenna infuscated or dark brown, subapical segments very short, more rounded laterally and second metasomal tergite dark brown or largely so (Figure 29H l.c.); [OOL 3.3–5.0 times diameter of posterior ocellus (Figure 29B l.c.); about 6 subapical antennal segments of ♀ moniliform and minute (Figure 29E l.c.); triangular prolongation of hypopygium of ♀ rather slender and about as long as second segment of hind tarsus (Figure 29I l.c.)] ································································································*P. sponsa* Ji & Chen, 2002

- Maximum width of pterostigma 0.9–1.0 times length of vein 3-SR; head yellow or with yellowish and dark brownish areas (Figure 4G–I); mesosoma more or less dark brown or blackish; POL as long as diameter of posterior ocellus; antenna brown and subapical segments less rounded, slightly longer; second tergite laterally dark brown; [apical lamella of carapace medially about half as long as 4th segment of hind tarsus; lower tooth of mandible larger and somewhat diverging from apical tooth] ································································································19

19 Scutellum and laterally second metasomal tergite black or dark brown; vein r of fore wing less angled with vein 3-SR; base of pterostigma with small yellow spot; [both sexes similarly dark colored] ································································································*P. diversa* (Walker, 1874)

- Scutellum and laterally second metasomal tergite brown (Figure 5J); vein r of fore wing distinctly angled with vein 3-SR (Figure 5A,B); pterostigma with comparatively large yellow basal patch (Figure 5B); [female with at most middle lobe of mesoscutum dark brown (Figure 5J); male meso- and metasoma largely black] ································································································*P. subdiversa* sp. nov.

Materials examined. Holotype, ♀, China: Zhejiang, Mt. Fengyang, 7 July 2007, Lanlan Zhu leg., (ZJUH No. 202402509). Paratypes, 1♀, China: Zhejiang, Mt. Fengyang, 28 July 2007, Lanlan Zhu leg., (ZJUH No. 202502655); 2♀♀, China: Shaanxi, Danfeng, 10 August 2014, light trap, (ZJUH Nos. 202502555, 202402556); 1♂, China: Zhejiang, Mt. Qingliang, 30 June 2007, light trap, (ZJUH No. 202502650).

Description. Holotype. ♀ (Figure 5). Length of body 4.8 mm, fore wing 4.0 mm.

Color. Brownish yellow (including humeral plate); stemmaticum black, head with mixture of yellowish and dark brownish areas (Figure 5G–I); clypeus largely brownish yellow; parastigma and veins of fore wing mainly yellowish brown; vein 1-R1 of fore wing basally yellow and remainder brown; apical half of hind femur and of middle and hind tibiae more or less darkened; remainder of legs pale yellowish or ivory; antenna apically, second and third tergites laterally darkened (Figure 5D,E); pterostigma (except large pale base) dark brown; patch below pterostigma infuscated; vein 1-M of fore wing mainly brown.

Head (Figure 5C,F–I) Width 1.4× median length in anterior view (Figure 5G) and part of head above eye in lateral view 0.4× height of eye (Figure 5H); antenna with 23 segments and 1.3× as long as fore wing, six subapical antennal segments moniliform, narrowed basally (Figure 5C), third, fourth, and penultimate segments 3.4, 3.0, and 1.6× as long as wide in lateral view; area of stemmaticum finely granulate; OOL: diameter of posterior ocellus: POL = 22:5:3; eye moderately protruding, about as long as temple in dorsal view (Figure 5I); frons with some curved striae medially, rather finely rugose laterally; vertex finely rugose; temple largely with curved rugae; face finely rugose and without distinct median ridge; clypeus finely punctate, shiny and about as wide as minimum width of face, intertentorial distance 1.8× minimum width between clypeus and eye, clypeus with three medium-sized acute teeth medio-ventrally (Figure 5G); eye in lateral view 1.1× as wide as temple (Figure 5H); in anterior view, height of eye 0.65× minimum width of face; upper condyle of mandible below lower level of eyes (Figure 5G); malar space 0.6× as long as basal width of mandible; lower tooth of mandible 0.4× length of apical tooth, robust and diverging (Figure 5F); head moderately emarginate medio-posteriorly (Figure 5I).

Mesosoma (Figure 5J). Length 1.5× its width in lateral view; side of pronotum mainly crenulate-rugose, shiny; mesoscutum coriaceous (lateral lobes) or densely rugulose (middle lobe, medio-posteriorly rugose) and rather dull, densely setose; notauli weakly indicated, rugose-crenulate; scutellar sulcus rather narrow and with eight carinae (Figure 5J); scutellum triangular, flattened, finely rugulose; metanotum with long median carina anteriorly and no tooth protruding posteriorly; propodeum coarsely reticulate-rugose, with irregular transverse carina, no median carina, latero-posteriorly distinctly tuberculate (Figure 5J).

Wings (Figure 5B). Fore wing 2.5× as long as its maximum width; length of 1-R1 1.3× as long as pterostigma; r issued rather far beyond middle of pterostigma and 0.4× as long as 3-SR; distance between 1-R1 and wing apex 0.3× 1-R1; 2-SR nearly straight and diverging from posterior margin of pterostigma (Figure 5B); SR1 straight or nearly so; parastigma large; m-cu slightly postfurcal; 1-CU1 0.2× as long as vein 2-CU1, cu-a weakly inclivous and about as long as 3-CU1; r:3-SR:SR1 = 10:23:79; 2-SR:3-SR:r-m = 36:23:14; 2-M weakly curved (Figure 5B).

Legs (Figure 5K). Hind femur rather shiny, very finely sculptured, 4.2× as long as wide and subparallel-sided submedially; hind tibia swollen (Figure 5K); middle tibia with small whitish blister; hind coxa superficially granulate and shiny.

Metasoma (Figure 5D,E,K). Carapace oval in dorsal view, 1.8× as long as wide and 1.1× as long as mesosoma; first and second tergites coarsely and densely longitudinally rugose; second suture narrow; third tergite 1.35× as long as second tergite and laterally curved, distinctly convex in lateral view, in dorsal view rounded, densely and rather coarsely rugose and medio-posteriorly ventral lamella shallowly concave in posterior view; ovipositor sheath rather narrow (Figure 5K) and parallel-sided, with some rather long setae; acute prolongation of hypopygium wide and short, its lateral sides subequal to its basal width.

Variation. Length of fore wing of female 3.3–4.0 mm, of male 3.2 mm; third tergite 1.3–1.6× as long as second tergite, curved laterally; POL 0.6–0.8× as long as diameter of posterior ocellus; eye in lateral view 0.8–1.1× as wide as temple; vein 2-SR of fore wing straight to moderately curved; vein cu-a of fore wing weakly to strongly inclivous.

Male. Meso-and metasoma and tegula black, but head mainly yellowish brown; antenna (except scape and pedicel) and parastigma dark brown; vein 2-CU1 of fore wing twice as long as vein 1-CU1; metasomal carapace elliptical in dorsal view, 2.1× as long as wide.

Host. Unknown.

Distribution. China (Shaanxi, Zhejiang).

Etymology. Named after *P. diversa* but is less diversely colored; ‘*sub*’ is Latin for ‘under, less’.

#### 3.3.5. *Phanerotoma trunculata* Fang, Luo and van Achterberg, sp. nov. (Figure 6)

Zoobank: urn:lsid:zoobank.org:act:5DA4527A-5B20-4096-A1AB-8A6D78DE32AE

**Figure 6:**
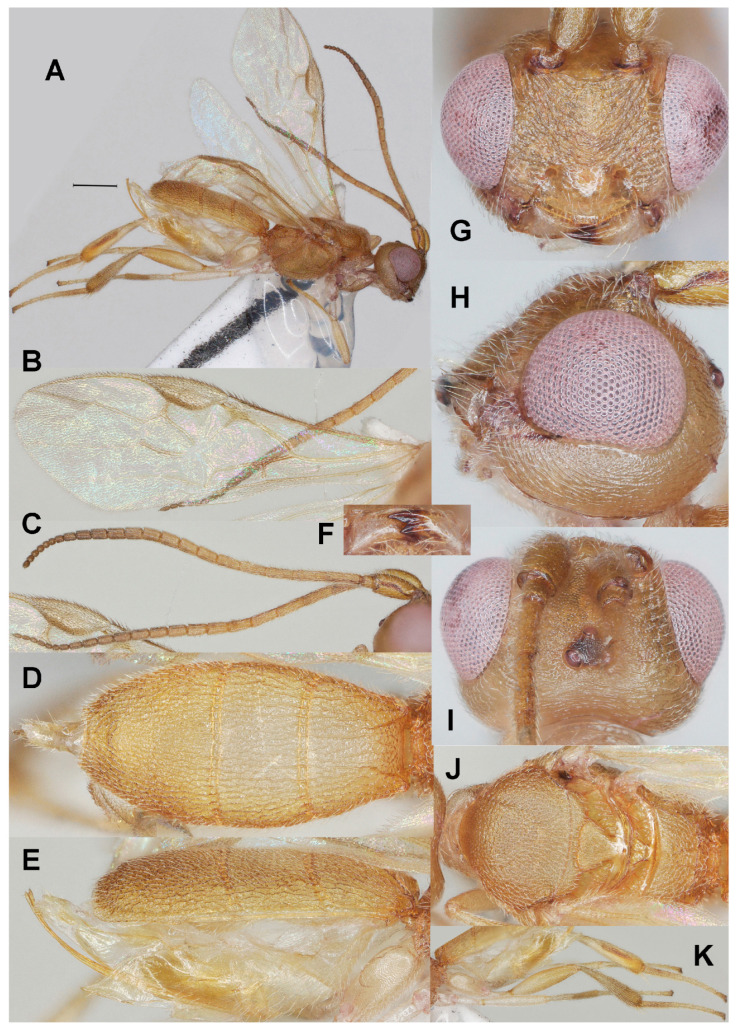
*Phanerotoma trunculata* sp. nov., holotype, ♀. (ZJUH No. 202500208) (**A**) Habitus, lateral aspect. (**B**) Fore wings. (**C**) Antenna, lateral aspect. (**D**) Metasoma, dorsal aspect. (**E**) Metasoma, lateral aspect. (**F**) Mandible, ventral aspect. (**G**) Head, anterior aspect. (**H**) Head, lateral aspect. (**I**) Head, dorsal aspect. (**J**) Mesosoma, dorsal aspect. (**K**) Hind leg, lateral aspect. Scale bar: 1.0 mm.

Diagnosis. Clypeus without ventral teeth, only with narrow truncate lamella ventrally (Figure 6G); five subapical antennal segments of ♀ moniliform and narrowed basally (Figure 6C); temple convex dorsally in lateral view (Figure 6H); malar space 0.5× as long as basal width of mandible (Figure 6H); vein 2-SR of fore wing slightly sinuate and diverging from posterior margin of pterostigma (Figure 6B); length of 1-R1 of fore wing 1.3× as long as pterostigma (Figure 6B); lateral sides of third tergite of metasoma in dorsal view curved and rounded posteriorly (Figure 6D,E); apical prolongation of hypopygium of ♀ acute in lateral view, rather short and lateral sides about equal to its basal width (Figure 6E); in lateral view metasomal carapace rather flat apically (Figure 6E), medio-posteriorly only lamella shallowly concave in posterior view.

Runs in the key to Chinese *Phanerotoma* species by Luo et al. [17] to couplet 4 and can be inserted as follows:

39 Vein 3-SR of fore wing 1–2 times as long as vein r (Figure 13G in Luo et al. [17]); vein 2-SR straight or slightly bent, vein SR1 straight or nearly so (Figure 24F l.c.); second submarginal cell rather narrow distally (Figure 12G l.c.), but wider in *P. trunculata* (Figure 6B) ································································································40a

- Vein 3-SR of fore wing 4–5 times longer than vein r (Figure 8F l.c.); vein 2-SR of fore wing distinctly curved and vein SR1 bent; second submarginal cell of fore wing rather wide distally (Figure 13G l.c.) ································································································41

40a Vein cu-a of fore wing subvertical (Figure 6B); lower tooth robust and comparatively long in respect to upper tooth (Figure 6F); head rather squarish in dorsal view (Figure 6I); [clypeus without minute teeth, only with small truncate medio-apical plate (Figure 6G); eye comparatively large (Figure 6H)] ································································································*P. trunculata* sp. nov.

- Vein cu-a of fore wing strongly inclivous (Figures 12A and 24F in Luo et al. [17]); lower tooth more slender and shorter in respect to upper tooth (Figures 12L and 24K l.c.); head less squarish in dorsal view (Figures 12B and 24B l.c.) ································································································40

Materials examined. Holotype, ♀, China: Zhejiang, Jieshoudao, 8 May 2008, light trap, (ZJUH No. 202500208).

Description. Holotype. ♀ (Figure 6). Length of body 3.3 mm, fore wing 2.8 mm.

Color. Brownish yellow; stemmaticum black; apex of antenna and mandible darkened; inner side of hind tibia with dark brown stripe (Figure 6A); pterostigma (except basal patch) and veins 1-CU1 and cu-a of fore wing infuscated; wing membrane subhyaline.

Head (Figure 6C,F–I) Width 1.3× median length in anterior view (Figure 6G) and part of head above eye in lateral view 0.3× height of eye (Figure 6H); antenna with 23 segments and about as long as fore wing; five subapical antennal segments narrowed basally, moniliform (Figure 6C), third, fourth and penultimate segments 3.6, 3.2 and 1.3× as long as wide in lateral view; area of stemmaticum mainly granulate; OOL: diameter of posterior ocellus: POL = 15:5:6; eye protruding, 3.0× temple in dorsal view (Figure 6I); frons with some curved rugae medially, rugose lateral with oblique rugae and without median carina; vertex finely granulate and posteriorly finely transversely rugulose-striate; temple largely with curved striae; face rugose (Figure 6G); clypeus mostly smooth, superficially punctulate, shiny and 0.8× as wide as minimum width of face, intertentorial distance 1.6× minimum width between clypeus and eye, clypeus largely smooth, sparsely punctate, with medium-sized setae and medio-ventrally with subtruncate lamella, without teeth (Figure 6G); eye in lateral view 1.8× as wide as temple (Figure 6H); in anterior view height of eye 0.9× minimum width of face; upper condyle of mandible near lower level of eyes (Figure 6G); malar space 0.5× as long as basal width of mandible; lower tooth of mandible 0.5× length of apical tooth, robust (Figure 6F); head rather squarish, hardly emarginate medio-posteriorly (Figure 6I).

Mesosoma (Figure 6J). Length 1.6× its width in lateral view; side of pronotum mainly crenulate medially, and remainder granulate, shiny; mesoscutum mainly densely rugulose (but medio-posteriorly rugose) and with satin sheen, densely setose; notauli weakly indicated, rugose; scutellar sulcus rather narrow and with eight carinulae (Figure 6J); scutellum triangular, flattened, granulate, but posteriorly superficially sculptured; metanotum with rather long median carina anteriorly and no tooth posteriorly; propodeum rather coarsely rugose, with irregular transverse carina, no median carina, latero-posteriorly weakly tuberculate.

Wings (Figure 6B). Fore wing 2.9× as long as its maximum width; length of 1-R1 1.3× as long as pterostigma; r issued far beyond middle of pterostigma and 0.5× as long as 3-SR; distance between 1-R1 and wing apex 0.2× 1-R1; 2-SR nearly straight and diverging from posterior margin of pterostigma (Figure 6B); SR1 straight or nearly so; parastigma large; m-cu interstitial; 1-CU1 0.6× as long as vein 2-CU1, cu-a subvertical and about as long as 3-CU1; r:3-SR:SR1 = 6:11:61; 2-SR:3-SR:r-m = 26:11:8; 2-M evenly curved (Figure 6B).

Legs (Figure 6K). Hind femur rather shiny, very finely sculptured, 3.7× as long as wide and widened submedially; hind tibia swollen (Figure 6K); middle tibia with small whitish blister; hind coxa superficially granulate and shiny.

Metasoma (Figure 6D,E,K). Carapace elliptical in dorsal view, 2.0× as long as wide and 1.2× as long as mesosoma; first and second tergites coarsely and densely longitudinally rugose; second suture narrow; third tergite 1.6× as long as second tergite and laterally curved, apically rather flat in lateral view, in dorsal view rounded, densely and rather coarsely rugose and medio-posteriorly ventral lamella shallowly concave in posterior view; ovipositor sheath rather narrow (Figure 6E) and parallel-sided, with some rather long setae; acute prolongation of hypopygium wide and short, its lateral sides subequal to its basal width (Figure 6K).

Male. Unknown.

Host. Unknown.

Distribution. China (Zhejiang).

Etymology. Named after the narrow truncate ventral apex of clypeus (Figure 6G), from the Latin ‘*trunculus*’ for ‘small cut off’.

## 4. Discussion

The integrated taxonomic approach employed in this study, combining DNA barcodes with morphology, has proven effective in elucidating species boundaries within the parasitoid wasp genus Phanerotoma. Our analysis, based on a robust dataset of 134 sequences, reveals significant cryptic diversity and provides a refined framework for the genus’ systematics. The discrepancy in MOTU delimitation between the distance-based ABGD (19 MOTUs) and the phylogeny-aware bPTP (27 MOTUs) methods is noteworthy. The finer partition recovered by bPTP, which was subsequently supported by diagnostic morphological characters, underscores a critical consideration for species-level studies. ABGD, which identifies gaps in pairwise genetic distances, may merge recently diverged lineages or those with slowed mitochondrial evolution where a clear “barcoding gap” has not yet developed. In contrast, bPTP models speciation events directly on a phylogenetic tree, making it more sensitive to delineating clusters that represent putative species, even in the absence of a pronounced distance gap. Our finding that comparative morphology aligned more strongly with the bPTP output (Figure 1) validates this model’s utility for groups like Phanerotoma, where morphological differentiation can be subtle but phylogenetically informative. This result aligns with growing evidence that single-locus species delimitation benefits from incorporating tree-aware methods alongside distance-based approaches.

Nevertheless, the interpretation of these results is subject to certain methodological constraints. The calculation of intraspecific genetic distances was precluded for several putative species due to the limited availability of specimens (1–2 individuals). While this inherently reflects the natural rarity of some taxa, it also underscores the need for expanded sampling to fully characterize lineage-specific variation. Additionally, the haplodiploid sex-determination system typical of braconid wasps introduces a potential source of variance. The strictly maternally inherited COI barcode in males may exhibit unexpectedly high divergence if specimens are sourced from different populations than females, a factor requiring cautious interpretation in the absence of corroborating nuclear data or direct breeding associations.

In conclusion, by synergizing molecular and morphological evidence, this study unveils hidden diversity within *Phanerotoma*, establishing five well-defined new species and providing a robust reference for future research. To further solidify these findings, future work incorporating multiple nuclear genes will be essential for reconstructing a robust phylogeny, testing species monophyly, and elucidating divergence histories. This refined framework will significantly aid subsequent biodiversity assessments, studies of host–parasitoid interactions, and biogeographic investigations of this ecologically vital genus.

## Data Availability

The data presented in this study are available in this article.

## Supplementary Materials

The following supporting information can be downloaded at: https://www.mdpi.com/article/10.3390/insects17020219/s1, Table S1: information of terminal taxa of Phanerotoma molecular analysis; Table S2: MEGA-K2p-distance; Dataset S3: *Phanerotoma*.align.fasta; Figure S4: ABGD_results based on Dataset S3; Figure S5: PTP species delimitation results-ML Partition based on Dataset S3; Treefile S6: Maximum likelihood tree of *Phanerotoma* based on Dataset S3; Dataset S7: *Phanerotoma* + *Siniphanerotomella*.align.fasta; Treefile S8: Maximum likelihood tree of *Phanerotoma* + *Siniphanerotomella* based on Dataset S7.
