# Supplementary figures and images for: DNA Barcodes and Morphology Reveal Five New Species of Phanerotoma (Hymenoptera, Braconidae, Cheloninae) from China
Source: Insects. 2026 Feb 20;17(2):219. doi: 10.3390/insects17020219 (PMC12941929; doi:10.3390/insects17020219)

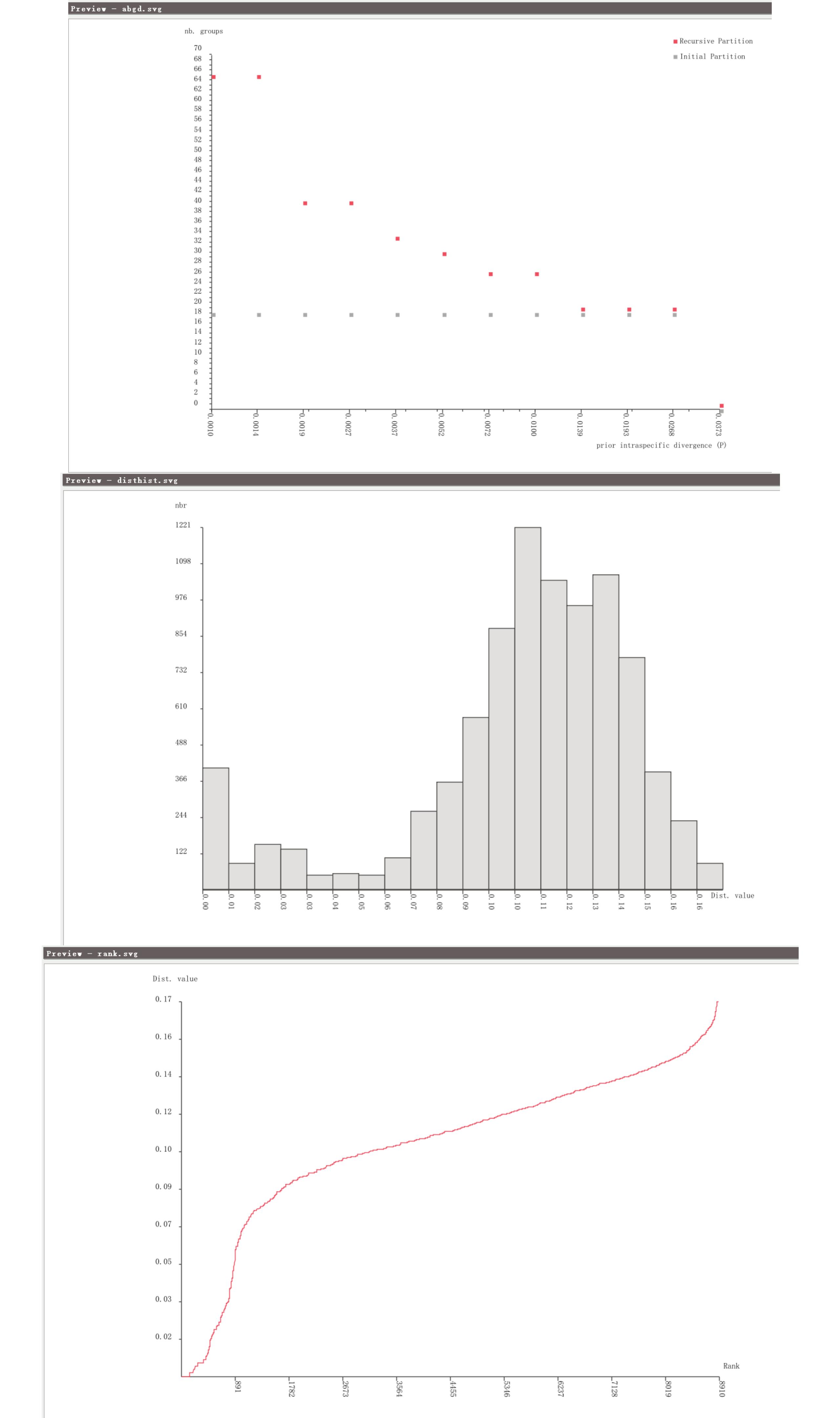

Supplement: Supplementary file 1 [file insects-17-00219-s001.zip › S4_ABGD_result.jpg]

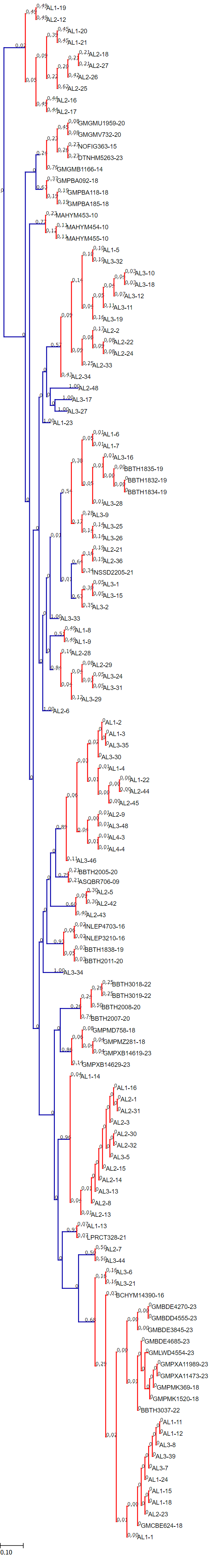

Supplement: Supplementary file 1 [file insects-17-00219-s001.zip › S5_PTP species delimitation results-MLPartition.svg.png]
